# Processing the fine-grained features of tactile textures involves the primary somatosensory cortex

**DOI:** 10.1162/imag_a_00341

**Published:** 2024-10-28

**Authors:** Giulia Esposito, Sylvie Nozaradan, Avgustina Kuzminova, Olivier Collignon, André Mouraux

**Affiliations:** Institute of Neuroscience, Université Catholique de Louvain, Woluwe-Saint-Lambert, Belgium; Institute of Research in Psychological Sciences, Université Catholique de Louvain, Louvain-La-Neuve, Belgium; School of Health Sciences, HES-SO Valais-Wallis, The Sense Innovation and Research Center, Lausanne and Sion, Switzerland

**Keywords:** EEG, vibrotactile, frequency-tagging, texture, somatosensory, S1

## Abstract

Dynamic tactile perception and discrimination of textures require the ability to encode and differentiate complex vibration patterns elicited at the level of the skin when sliding against a surface. Whether the primary somatosensory cortex (S1) can encode the fine-grained spectrotemporal features distinguishing textures remains debated. To address this question, electroencephalography (EEG) frequency-tagging approach was used to characterize responses to vibrotactile oddball contrasts between two textures. In a first session designed to identify the topographical distribution of responses originating from the hand and foot representations in S1, standard and deviant stimuli were pure sinusoidal vibrations differing in frequency and intensity. In a second session, standard and deviant stimuli were two different snippets of bandpass-filtered white noise matched in terms of intensity and average frequency content, but differing in terms of their complex spectrotemporal content. Using the S1 functional localizer, we showed that oddball responses to a spectrotemporal contrast follow the somatotopical organization of S1. Our results suggest that the encoding of fine-grained spectrotemporal features associated with different vibration patterns involves S1.

## 1 Introduction

During tactile exploration of a textured surface, minute and complex deformations of the skin activate mechanoreceptors located within cutaneous and subcutaneous structures. It is believed that two main codes underlie our ability to discriminate textures: a spatial code, whereby texture information is conveyed by the differential activation of mechanoreceptors having small receptive fields, and a temporal code mediated by differences in the temporal dynamics of activity generated within rapidly adaptive mechanoreceptors (Connor & Johnson, 1992;Connor et al., 1990;Hollins & Risner, 2000;Muniak et al., 2007). Indeed, sliding the finger against a fine-grained textured surface generates mechanical vibrations at the skin–surface interface that preferentially activate rapidly adapting mechanoreceptors types I and II (Bensmaïa & Hollins, 2005;Hollins et al., 2001). Therefore, the frequency content and time course of skin vibrations during exploration of natural textures may carry information about individual texture identity (Manfredi et al., 2014). More specifically, the intensity of vibrations recorded at the level of the hand and wrist during dynamic exploration of a textured surface was shown to carry information about texture roughness for both periodic and non-periodic textures, while their frequency content allowed discrimination of different samples of periodic textures (Delhaye et al., 2012). In primates, several studies have reported temporal encoding of skin vibrations and fine textures within vibration-sensitive mechanoreceptors, with millisecond-scale precision (Mackevicius et al., 2012;Weber et al., 2013). The temporal pattern of activation of rapidly adapting types I and II fibers has been shown to reliably reflect individual texture identity, and has thus been suggested to mediate fine texture perception (Weber et al., 2013). Other studies conducted in non-human primates have shown that millisecond-level temporal patterning of responses to natural textures is also represented at the cortical level, in the primary somatosensory cortex (S1) (Birznieks & Vickery, 2017;Harvey et al., 2013;Long et al., 2022). The cortical activity generated by high-frequency vibrations has been related to the envelope and the frequency content of skin vibrations elicited by surface scanning of the fingertip (Harvey et al., 2013).

In humans, scalp electroencephalography (EEG) can be used to non-invasively characterize brain responses to a sensory stimulus with a very fine time scale. However, measuring high-frequency cortical activity to high-frequency vibrations using scalp EEG is a challenging endeavor and, using pure sinusoidal mechanical vibrations, EEG responses have been previously shown to only be reliably recorded at stimulation frequencies below 100 Hz (Breitwieser et al., 2012;Srinivasan et al., 1990;Vialatte et al., 2009).

Besides the recording of event-related potentials (ERPs) which allow characterizing synchronized and transient cortical responses to the onset of single sensory events, EEG can also be used to characterize cortical activity induced by the periodic and selective modulation of a property of the sensory stimulus using the frequency-tagging approach (Lenc et al., 2021;Norcia et al., 2015;Nozaradan, 2014;Rossion, 2014). In this approach, periodic modulation of a sensory feature elicits a periodic variation in cortical activity, which is expected to project at the frequency of the periodic modulation and its harmonics in the EEG spectrum (Nozaradan, 2014;Rossion, 2014). Therefore, an advantage of the frequency-tagging approach is the fact that it allows specific characterization and quantification of the elicited responses as they are constrained within exact narrow frequency bands determined by the rate at which the sensory feature is modulated (Norcia et al., 2015;Regan, 1966,^1989^). Using this approach,Moungou et al. (2016)were able to isolate and describe, in humans, cortical activity related to the processing of natural fine-grained textures. To do so, they periodically modulated the*envelope*of high-frequency vibrations elicited at the fingertip by introducing a small 3 Hz sinusoidal vertical displacement of the textured surface while it was concurrently sliding against the finger. Besides showing that the EEG frequency-tagging approach was able to capture stimulus-related cortical activity tagged at the frequency of this vertical displacement, they also reported that the magnitude of these responses was related predominantly to the magnitude of the high-frequency vibrations recorded at the fingertip. Furthermore, the topographic pattern of the periodic texture-related activity was contralateral to the stimulated hand, suggesting an involvement of S1 (Moungou et al., 2016). Classification of textures differing in their levels of roughness has also been recently achieved using EEG, indexed by variations in the total power of Mu- (8–15 Hz) and beta-band (16–30 Hz) oscillations (Eldeeb et al., 2020). Importantly, texture classification accuracy was highest around electrodes contralateral to the stimulation site (Eldeeb et al., 2020), further supporting tactile texture representation within S1. However, whether the processing and discrimination of different fine-grained textures are implemented within S1, or whether they require higher cortical areas remains unknown. In a recent investigation, decreases in alpha-band power were observed over bilateral sensorimotor areas in response to both smooth-to-rough and rough-to-smooth changes in textures in conditions of active, dynamic touch (Henderson et al., 2023), possibly suggesting involvement of areas beyond S1. Some neuroimaging studies have reported texture-selective activity in S1 (Lederman et al., 2001) as well as in the secondary somatosensory cortex (S2) and the insula (Kitada et al., 2005;Simões‐Franklin et al., 2011). Neuroimaging studies have reported an involvement of S2 and the insula in the categorization of textures based on high-level texture characteristics, such as perceived roughness, while activity within S1 would be more closely associated with low-level differences in the physical characteristics of textures, such as their spatial period (Eck et al., 2016).

With some exceptions (Henderson et al., 2022,^2023^;Moungou et al., 2016), previous studies have largely relied on simple synthetic stimuli, such as raised dots or ridges, displaying very regular spatial characteristics. Such physical features poorly relate to the complex patterns of vibrations that are elicited upon scanning of natural textures. At the same time, previous work has largely focused on higher-level aspects of texture processing, for instance comparing rough and smooth textures. What thus needs to be further investigated is whether S1 is able to resolve minute variations in the frequency composition over time of vibrations that distinguish fine-grained textures, and, therefore, whether contrast-specific responses to spectrotemporal differences in vibrotactile input matching in terms of their overall intensity and spectral content involve S1.

Here we address this question using a variation of the EEG frequency-tagging approach referred to as periodic oddball stimulation. In this paradigm, a deviant or oddball stimulus periodically replaces a standard stimulus within a regular sequence repeated at a constant frequency (Norcia et al., 2015;Rossion, 2014;Rossion et al., 2015). The strength of the approach lies in the fact that it allows distinguishing EEG responses that are related to the processing of stimulus features that are shared across the standard and deviant stimuli from those related to processing the specific features distinguishing the standard stimulus from the deviant stimulus. Indeed, the first will appear in the EEG frequency spectrum as peaks at the frequencies corresponding to the base rate of stimulation—and its harmonics—whereas the second will appear as peaks at the frequencies corresponding to the rate of presentation of the deviant stimuli—and its harmonics (Nozaradan et al., 2017). The approach has been widely employed to characterize cortical processes involved in auditory (Nozaradan et al., 2017) and visual (Norcia et al., 2015;Rossion, 2014) discrimination. To date, no studies have implemented periodic oddball stimulation to investigate somatosensory processing of changes in vibrotactile stimulation.

The aim of this experiment was thus to explore whether it is possible to record EEG responses to fine-grained vibrotactile contrasts using the periodic oddball stimulation paradigm, and to examine whether these responses originate, at least in part, from the contralateral S1. In a first condition, the difference between the oddball and standard stimuli consisted in a low-level contrast in tactile vibration frequency and intensity. In a second condition, we implemented a higher-level fine-grained spectrotemporal contrast, where standard and oddball stimuli were complex bandpass filtered (40–200 Hz) white noise vibrations that did not differ in terms of their average intensity or average frequency content, but in the time course of their envelope and spectral content. Our main hypotheses were (1) that oddball EEG responses can be recorded to both frequency–intensity and spectrotemporal contrasts and (2) that the topography of the oddball responses to spectrotemporal contrasts would be consistent with activity originating at least partly from S1, based on recent evidence that fine-grained texture discrimination involves S1 (Moungou et al., 2016). To test the second hypothesis, we stimulated two body sites that would allow assessing whether the topography of oddball EEG responses would follow the somatotopical organization of S1: the index finger of the right hand, for which we expected to observe responses clearly lateralized to the contralateral hemisphere, and the sole of the right foot, for which we expected responses localized at central scalp electrodes (Dowman & Schell, 1999;Heid et al., 2020). While somatotopy has also been characterized in areas beyond S1 such as S2 and the insula using functional magnetic resonance imaging (fMRI) and magnetoencephalography (MEG) (Björnsdotter et al., 2009;Del Gratta et al., 2002;Ruben et al., 2001), hand versus foot somatotopy in these higher-order areas is not expected to generate marked differences in EEG scalp topography (Ruben et al., 2001).

## 2 Methods

### 2.1 Participants

Eighteen healthy participants (5 males, 13 females, aged 22–32 years) were recruited to participate in the study. Participants received information about the study and gave written informed consent. All participants confirmed they had no current or history of neurological or psychiatric disorders, and no loss of skin sensitivity at their hands or feet. The study was approved by the UCLouvain Ethics Committee. Data from one subject were excluded due to highly noisy EEG recordings. The final sample thus included 17 healthy participants (14 right handed; 1 left handed; handedness information missing from 2 participants).

### 2.2 Stimuli and apparatus

Vibrotactile stimuli were designed in MATLAB (version R2020b) and consisted of sequences of vibrotactile stimuli delivered according to an established (AAAAB) oddball pattern (Liu-Shuang et al., 2014;Nozaradan et al., 2017;Rossion, 2014). The base repetition frequency was 8 Hz. The frequency of the oddball B stimulus was 8/5 Hz = 1.6 Hz. For each trial, this pattern was looped 64 times, for a total of 40 s of periodic oddball vibrotactile stimulation. The rate of stimulation was chosen as, at 8 Hz, the 125 ms duration of individual stimuli was long enough to optimize contrast detection, but not so fast as to impair individual stimulus detection due to fusion effects, as vibrotactile stimuli modulated at frequencies up to 10–13 Hz are still perceived as individual events (Jurado et al., 2021;Park & Choi, 2011). Further, a relatively fast stimulation rate allowed presenting more stimuli in a short amount of time, thus optimizing signal-to-noise ratio. These 40-s sequences were delivered in separate trials to the right index finger or the right inner midfoot.

#### 2.2.1 Frequency–intensity contrast

In this first condition, individual A and B vibration stimuli were sine waves lasting 125 ms, with the amplitude of vibration linearly ramping up from 0 to 1 (full amplitude) and down from 1 to 0 during 15 ms at the beginning and end of each stimulus, respectively. The A stimuli had a frequency of 300 Hz and the B stimuli had a frequency of 200 Hz. These frequencies were chosen as responses to both A and B stimuli would be processed preferentially by rapidly adapting mechanoreceptors type II, responding maximally to frequencies in the 200–300 Hz range (Holmes & Tamè, 2023;Verrillo, 1985), and thought to largely contribute to the perception of fine textures (Bensmaïa & Hollins, 2005;Hollins et al., 2002). This condition was used to assess the feasibility of recording oddball responses to a low-level contrast in frequency and/or intensity of stimulation. Indeed, considering the frequency dependence of skin mechanoreceptors, changing the frequency of stimulation inevitably produces changes in the intensity of mechanoreceptor activation, and differential activation of different mechanoreceptor classes (Johansson & Vallbo, 1983). This condition was also used to obtain a topographic template of EEG responses to vibrotactile stimulation of the hand and foot, as described inSection 2.6.4.

#### 2.2.2 Spectrotemporal contrast

In this second condition, A and B stimuli were also of 125 ms duration, with 15 ms ramps up and down. The only difference with the first condition was that A and B stimuli were made of broadband noise instead of sine waves. For each trial, A stimuli consisted of 125-ms white noise, and B stimuli were generated by shuffling the amplitude values of noise A across time points. The two obtained white noise samples were then band-pass filtered between 40 and 200 Hz, to specifically stimulate within the preferred frequency band of rapidly adapting mechanoreceptors types I and type II (Johansson & Vallbo, 1983), and reduce potential confounding auditory stimulation elicited by high frequencies which would not concomitantly contribute to mechanoreceptor activation, which is typically observed at frequencies up to 1,000 Hz (in the case of rapidly adapting mechanoreceptors type II) (Bell et al., 1994;Jones & Smith, 2014). Eight different trials were generated using eight random samples of white noise (stimulus A) and their shuffled version (stimulus B). For both conditions, A and B stimuli were RMS normalized to equalize their total energy. The A and B stimuli were thus matched in terms of energy and average spectral content, but differed in terms of envelope and spectral time course. All stimuli were delivered using a vibrating device (Minishaker Type 4810, Bruel & Kjaer) connected to a power amplifier (Type 2718, Bruel & Kjaer). A curved metal plate (45 mm length, 22 mm width) was attached to the top of the actuator using a 10–32 UNF screw. To maintain intensity of stimulation perceptually similar in both conditions, the amplitude of the frequency/intensity contrast sequences was set to about half that of the white noise sequences, as determined by pilot experiments. The stimulation laptop was connected to a digital-to-analog converter (USB 6363, National Instruments), which delivered the analog output to the amplifier and sent triggers to the EEG recording system at the onset of each trial. A custom platform was built to ensure participants received stimulation in an optimal manner while being in a comfortable position and to minimize finger and foot movements. The vibrating device was placed inside the platform, and support pistons allowed to adjust the height of the device. For hand stimulation, participants rested their forearms on the platform, placing their right index finger on the vibrating plate. For foot stimulation, participants rested their right foot on the platform, placing their inner midfoot on the vibrating plate. The choice of location for foot stimulation was justified by previous studies reporting lower tactile perception thresholds for the inner midfoot compared with the big toe (Kekoni et al., 1989), which we reasoned would allow maximizing the EEG responses to foot stimulation.

### 2.3 Procedure

The experiment consisted of a total of 64 trials, divided into 4 blocks (2 for hand and 2 for foot stimulation). Within each block, 16 trials (8 frequency/intensity trials and 8 spectrotemporal trials) were randomly presented. During each trial, subjects listened to white noise played through headphones to cover any auditory noise generated by the vibrating device. To ensure participants did not hear the sequences, they were presented with one frequency/intensity and one spectrotemporal sequence without concomitant tactile stimulation, and the level of white noise was adjusted until participants reported not hearing the sequences. To avoid any order effects, the order of stimulation location (hand or foot) was counterbalanced across participants. Subjects were asked to pay attention to the stimuli and to report, at the end of each trial, whether they were able to detect only one type of vibration (no contrast) or more than one type of vibration (contrast).

Prior to the experimental session, participants were presented with two example sequences, one with no contrast (only 47 Hz vibrations) and one with contrast (where A = 47 Hz and B = 197 Hz), to make sure they familiarized themselves with the oddball contrasts.

### 2.4 EEG data acquisition

Participants were instructed to relax, avoid moving their head and body as much as possible, and fixate on a point in front of them. A 64 Ag–AgCl electrode cap (ANT) was used for EEG recordings and placed on the participants’ scalp according to the International 10/10 system. The signals were recorded using an average reference. Sample rate was set at 2,000 Hz and impedances were kept below 10 kΩ.

### 2.5 Spectrotemporal stimuli envelope dissimilarity analysis

For each of the eight spectrotemporal sequences, the A and B stimuli were designed to be matched in terms of total energy and average frequency content. However, they could differ both in terms of their temporal envelope (envelope contrast) or their fine-grained spectral content over time (spectrotemporal contrast). In order to investigate whether the oddball EEG responses to these stimuli could have been driven predominantly by differences in the time course of the envelopes of A and B white noise stimuli, a dissimilarity index was computed for each of the eight trials as the sum of the absolute point-by-point difference between the envelope of the A stimulus and its corresponding B stimulus (obtained by means of a Hilbert transformation of the A and B stimuli) in the time domain. As shown inFigure 1C, one of the eight sequences used (spectrotemporal sequence #2) exhibited a strong dissimilarity in envelope as compared with all other sequences.

**Fig. 1.**
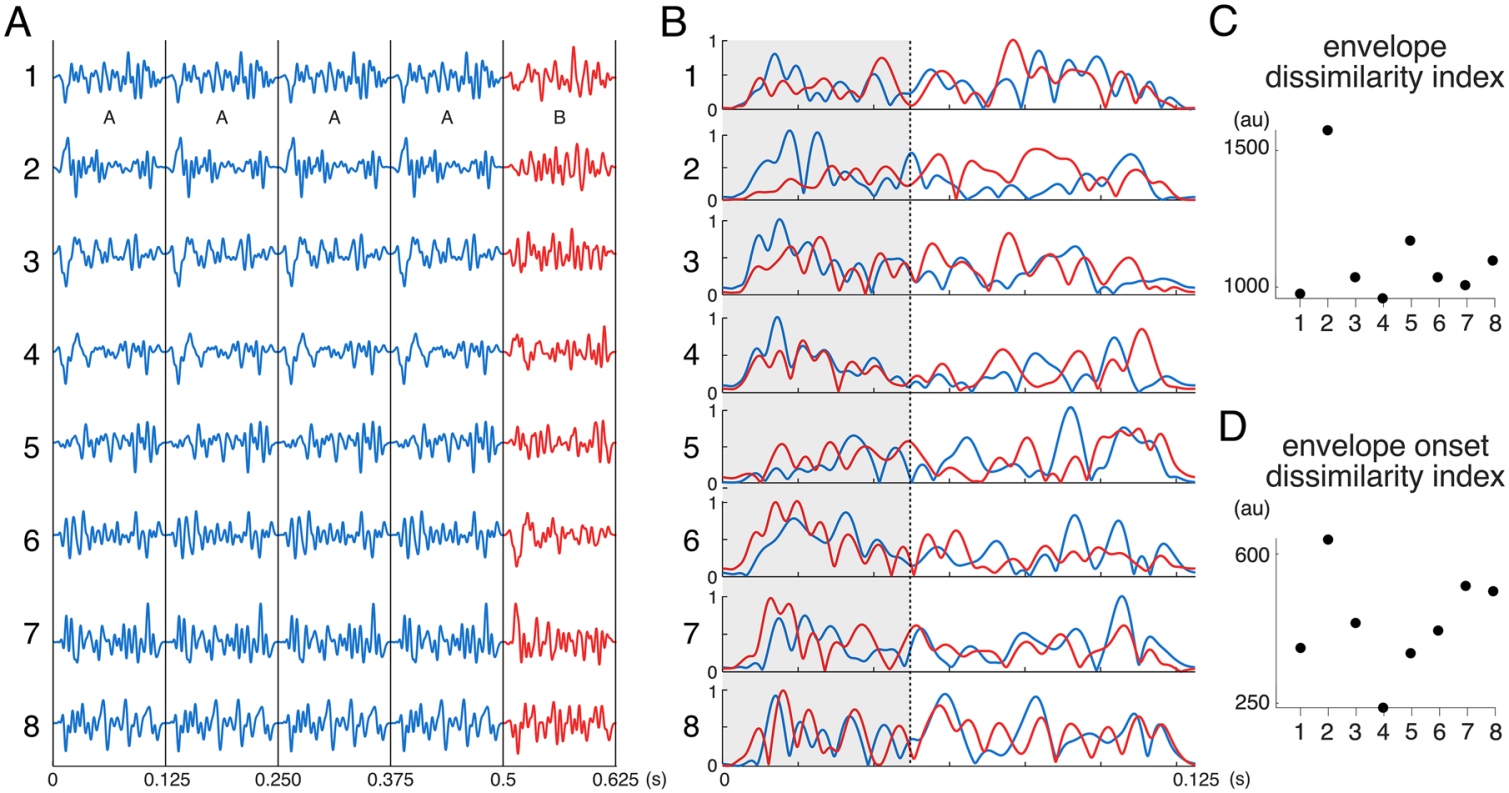
(A) Time course of spectrotemporal A and B stimuli 1–8 (x-axis: time in seconds.; y-axis: amplitude (AU), where 1 is maximum amplitude output). (B) Time course of the envelopes of spectrotemporal A and B stimuli 1–8 (x-axis: time in seconds.; y-axis: amplitude (AU), where 1 is maximum amplitude output). (C) Index of envelope dissimilarity between A and B stimuli 1–8. (D) Index of envelope onset (first 50 ms) dissimilarity between A and B stimuli 1–8.

The EEG analyses reported in the manuscript were performed excluding responses to the highly dissimilar spectrotemporal sequence 2. Results obtained using the dataset including spectrotemporal sequence 2, and additional analyses to explore whether oddball responses to spectrotemporal contrasts could have been driven by differences in the envelope of the A and B stimuli are reported asSupplementary Materials.

### 2.6 EEG Analysis

#### 2.6.1 Preprocessing

EEG data preprocessing and analysis steps were performed using the Letswave (version 6;https://www.letswave.org/) Matlab toolbox for EEG signal processing. First, a DC removal and linear detrend were applied on the continuous EEG traces to remove drifts in the signals. Next, a bandpass Butterworth filter (4th order) was applied between 0.1 and 40 Hz. To facilitate data handling, EEG traces were then downsampled to 500 Hz and segmented from 0 to 40 s relative to trial onset. Occasional noisy channels were identified visually and replaced by the average of the signals recorded at the three closest neighboring electrodes. A total of 12 channels were interpolated across 6 subjects. To remove ocular artifacts (eye blinks and eye movements), an independent component analysis (ICA) was performed using the RUNICA algorithm (constrained to 63-N independent components, where N corresponded to the number of interpolated channels). Independent components capturing electrooculographic activity were identified visually (based on their time course and topography) and removed. Finally, trials presenting remaining large-amplitude artifacts were automatically rejected using an amplitude criterion of 500 µV. A total of 47 trials were removed (14 and 9 trials for the frequency/intensity condition at the hand and foot; 19 and 5 trials for the spectrotemporal condition at the hand and foot) across 14 subjects. Finally, signals were re-referenced to the average of all scalp channels.

#### 2.6.2 Frequency domain analysis

For each participant, stimulation type and stimulation site, EEG signals were averaged across trials in the time domain to reduce the contribution of signals that were not time locked with the trial onsets. A fast Fourier Transform (FFT) was applied to obtain amplitude spectra with a resolution of 0.025 Hz (i.e., 1/40 s), which allowed further analysis of responses at the frequencies of interest and their harmonics. To evaluate the periodic EEG response to the base and oddball stimuli, a baseline subtraction was applied to the single-subject amplitude spectra at each scalp channel by removing, at each frequency bin, the average of the 24 neighboring frequency bins (12 on each side, excluding bins immediately before and after the bin of interest). The baseline subtraction procedure minimizes the contribution of background EEG activity and noise to the activity at frequency bands of interest. In the absence of a periodic EEG response at the frequency of interest, baseline-subtracted amplitudes should tend toward zero (Chemin et al., 2014;Freeman et al., 2003;Mouraux et al., 2011;Nozaradan et al., 2011,^2017^).

#### 2.6.3 Significance testing of base and oddball EEG responses

For each participant, condition, and stimulation site, the baseline-subtracted frequency spectra were averaged across all scalp channels.

To assess whether a periodic response was elicited at the base frequency, baseline-corrected amplitudes at the base frequency and its harmonics were averaged up to 40 Hz (8, 16, 32, and 40 Hz). The resulting average values were tested against zero using a one-sample t-test (right tailed), separately for each stimulation type and stimulation site. Similarly, to test for the presence of an oddball response, baseline-corrected amplitudes at the oddball frequency and harmonics were averaged for frequencies up to 40 Hz—excluding frequencies corresponding to the base frequency and its harmonics—and tested against zero. Aggregation of harmonics is recommended as it allows to more effectively compare responses across conditions because different EEG response time courses may lead to different levels of amplitude at different harmonics, and inclusion of higher harmonics allows a better characterization of overall responses (Retter et al., 2021).

#### 2.6.4 Topographic analysis of somatotopy

Next, we aimed to investigate our central hypothesis that a substantial part of the oddball EEG response to spectrotemporal contrasts originates from S1. Because a periodic intensity modulation of vibrotactile input may be expected to generate a strong periodic variation in S1 activity, the base responses to the frequency/intensity sequences can be expected to predominantly reflect activity originating from S1 (Colon et al., 2012,^2014^). Indeed, fMRI and MEG studies have shown that vibrotactile stimulation primarily activates the contralateral S1 (Kim et al., 2015;Stippich et al., 1998;Tuunanen et al., 2003), and that the magnitude of S1 responses increases with increasing amplitude of vibrotactile stimuli under task-free conditions (Nelson et al., 2004). Most importantly, and notwithstanding the fact that the vibrotactile stimuli are expected to generate activity beyond the contralateral S1, the differences in the scalp topography generated by vibrotactile stimulation of the hand versus the foot may be expected to predominantly result from the somatotopical organization of S1. The EEG responses to the base repetition of frequency–intensity contrasts were thus used to generate two weighted channel templates, one built using the base response during hand stimulation to capture the topographical distribution of activity originating from the hand representation within S1, the other built using the base response during foot stimulation to capture the topographical distribution of activity originating from the foot representation within S1. To construct each of the two templates, we first identified the base frequency harmonics exhibiting amplitude values significantly greater than zero when applying the frequency/intensity stimulus to the hand or foot, respectively (t-test against zero using the frequency spectra averaged across all scalp channels). Then, amplitude values at each channel were averaged across significant harmonics. Negative amplitude values were set to 0, and the obtained channel template was divided by its sum across channels, such that the sum of the template would be equal to 1. In this way, a weight was assigned to each channel, depending on the relative amplitude of the base response across channels. This procedure allowed us to assess S1 somatotopy while maintaining the spatial information provided by all electrodes, rather than by using an*a priori*selection, and at the same time by accounting for the relative contribution of each electrode (represented by the weight assigned in the templates) to the observed responses following hand and foot stimulation. Indeed, a similar topographical distribution of responses at both stimulation sites would lead to similar weights being assigned to the same electrodes. As such, even in the presence of differences in amplitudes following hand and foot stimulation, multiplying the amplitude of responses of one stimulation site by the weights of the two templates would lead to very similar results. However, in the presence of topographical differences at the two stimulation sites, different weights would be assigned to the same electrodes in the two templates, and multiplying the amplitude of responses of one stimulation site by the weights of the two templates would allow us to capture such differences of spatial distribution.

For each participant and stimulation site, the EEG response to the base frequency of the spectrotemporal contrast (average of baseline-subtracted amplitudes at base frequency and harmonics up to 40 Hz) and the EEG response to the oddball frequency of the spectrotemporal contrast (average of baseline-subtracted amplitudes at the oddball frequency and its harmonics up to 40 Hz, excluding frequencies corresponding to the base harmonic frequencies) were multiplied with each of the two weighted channel templates. The obtained weighted amplitudes were then averaged across all channels, yielding four values for each participant and stimulation site, corresponding to the base and oddball EEG responses weighted by the hand and foot templates, respectively.

Finally, the magnitudes of the responses elicited by hand versus foot stimulation weighted by the hand versus foot template were compared using a 2 by 2 repeated-measures ANOVA with the factors “stimulation site” (hand vs. foot stimulation) and “channel template” (hand template vs. foot template).

Our hypothesis was that, in the presence of S1 somatotopy, the repeated-measures ANOVA would reveal a significant interaction between the two factors, because the amplitude of the response elicited by stimulation of the hand weighted by the hand template would be significantly greater than the amplitude of that same response weighted by the foot template; whereas the response elicited by stimulation of the foot would be greater when weighted by the foot template as compared with the hand template. When significant, post hoc comparisons using paired-sample t-tests were used to compare, for each stimulation site, the amplitudes of the oddball response weighted by the hand template versus the foot template.

## 3 Results

### 3.1 Base and oddball EEG responses

**Fig. 2.**
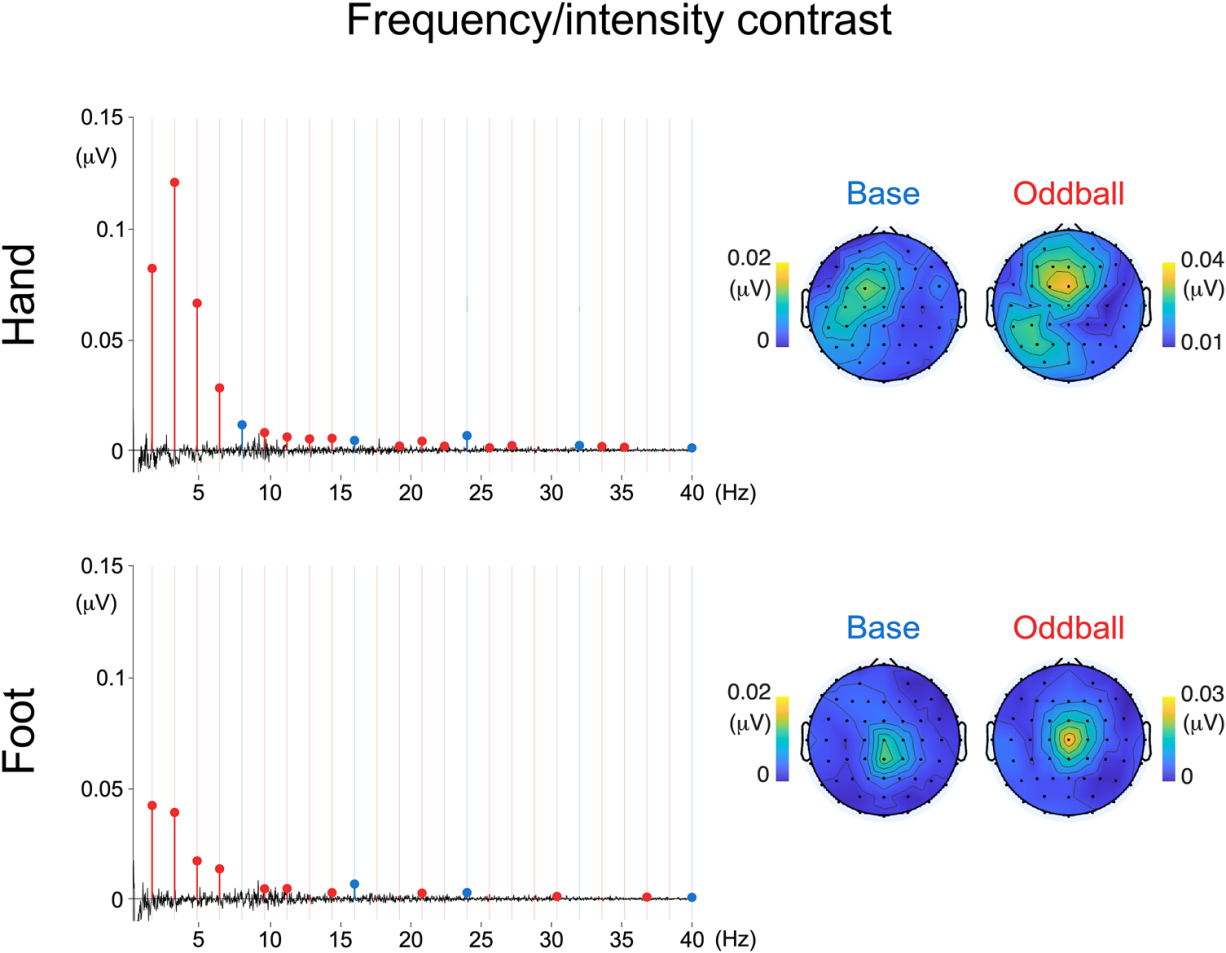
Baseline-subtracted EEG frequency spectra in the frequency/intensity contrast condition across hand and foot stimulation, averaged across participants and EEG channels. Harmonics are color coded, with base responses depicted in blue, and oddball responses depicted in red. Filled dots depict harmonics with amplitude values significantly higher than zero (t-test, right-tailed). Vertical bars represent expected base and oddball frequencies. Topographical plots represent signal amplitude averaged across significant harmonics.

Both for hand stimulation and foot stimulation, significant base responses (averaged across all harmonics up to 40 Hz) were observed for the frequency/intensity contrast condition (hand: mean = 0.005 µV; SD = 0.007 µV; p = 0.004; foot: mean = 0.003 µV; SD = 0.004 µV); p < 0.001) (Fig. 2).

**Fig. 3.**
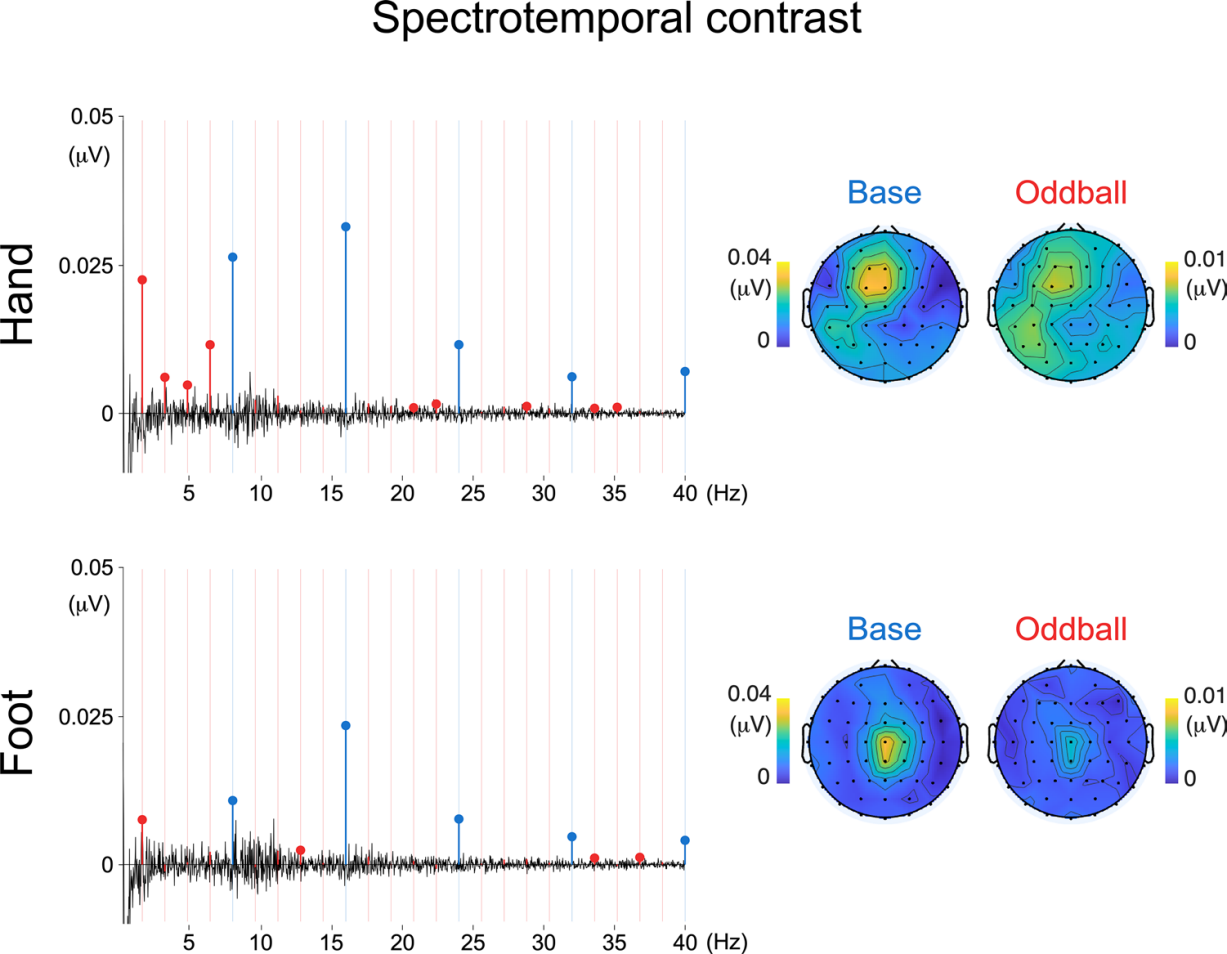
Baseline-subtracted EEG frequency spectra in the spectrotemporal contrast condition across hand and foot stimulation, averaged across participants and EEG channels. Harmonics are color coded, with base responses depicted in blue and oddball responses depicted in red. Filled dots depict harmonics with amplitude values significantly higher than zero (t-test, right-tailed). Vertical bars represent expected base and oddball frequencies. Topographical plots represent signal amplitude averaged across significant harmonics.

Significant base responses (averaged across all harmonics up to 40 Hz) were also observed the spectrotemporal contrast condition (hand: mean = 0.016 µV; SD = 0.015 µV; p < 0.001; foot: mean = 0.010 µV; SD = 0.008 µV; p < 0.001) (Fig. 3).

All base frequencies up to 40 Hz exhibited baseline-subtracted amplitude values significantly greater than zero in at least one stimulation condition or stimulation site (Figs. 2and3).

Similarly, both for hand and foot stimulation, significant oddball responses (averaged across all harmonics, excluding base frequencies) were observed both for the frequency/intensity contrast (hand: mean = 0.017 µV; SD = 0.005 µV; p < 0.001; foot: mean = 0.007 µV; SD = 0.003 µV; p < 0.001) and for the spectrotemporal contrast (hand: mean = 0.003 µV; SD = 0.002 µV; p < 0.001; foot: mean = 0.001 µV; SD = 0.002 µV; p = 0.013). Oddball harmonics from 1.6 to 14.4 Hz and from 19.2 to 36.8 Hz exhibited amplitudes significantly greater than zero in at least one stimulation type (frequency/intensity or spectrotemporal contrast), at one or both stimulation sites (hand or foot) (Fig. 2).

**Fig. 4.**
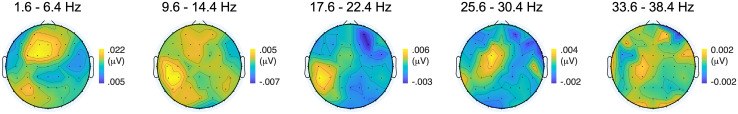
Topographical plots of the oddball EEG responses to the spectrotemporal contrast averaged across clusters of oddball harmonics (1.6–6.4 Hz; 9.6–14.4 Hz; 17.6–22.4 Hz; 25.6–30.4 Hz; 33.6–38.4 Hz).

Visual inspection of the topographical distribution of the periodic EEG responses showed that, across both types of contrasts, both the base response and the oddball response were localized over central and parietal scalp electrodes contralateral to the stimulated limb. In contrast, for base and oddball responses to foot stimulation, the responses to both types of stimuli were localized over central scalp electrodes.Figure 4shows the topography of the responses to hand stimulation using the spectrotemporal contrasts across clusters of oddball harmonics (1.6–6.4 Hz; 9.6–14.4 Hz; 17.6–22.4 Hz; 25.6–30.4 Hz; 33.6–38.4 Hz), which remained stable and contralateral to the stimulated hand.

### 3.2 Topographic test for somatotopy

Significant harmonic frequencies that were averaged to generate the weighted channel templates were 8, 16, 24, 32, and 40 Hz for hand stimulation, and 16, 24, and 40 Hz for foot stimulation. The 10 channels showing maximum amplitudes in the hand template were FC1, FCz, C3, C1, FC3, CP3, F1, Fz, Cz, and CP5. For the foot template, they were CPz, Cz, CP2, C2, Pz, P1, CP1, FCz, FC2, and POz. The templates are shown inFigure 4.

#### 3.2.1 EEG response to the base repetition of spectrotemporal contrasts

The repeated-measures ANOVA showed a significant interaction between stimulation site (hand vs. foot) and channel template (hand vs. foot template), indicating that the topography of the base response to the spectrotemporal contrast depended on the site of stimulation (F = 14.666, p = 0.001, η^2^= 0.031). Post hoc t-tests (one sided) showed S1 somatotopy: the base response to stimulation of the hand was greater when weighted with the hand template as compared with the foot template (p = 0.003, Cohen’s d = 0.784), whereas the base response to stimulation of the foot was greater when weighted with the foot template than when weighted with the hand template (p = 0.002, Cohen’s d = 0.823).

#### 3.2.2 Oddball EEG response to the spectrotemporal contrasts

**Fig. 5.**
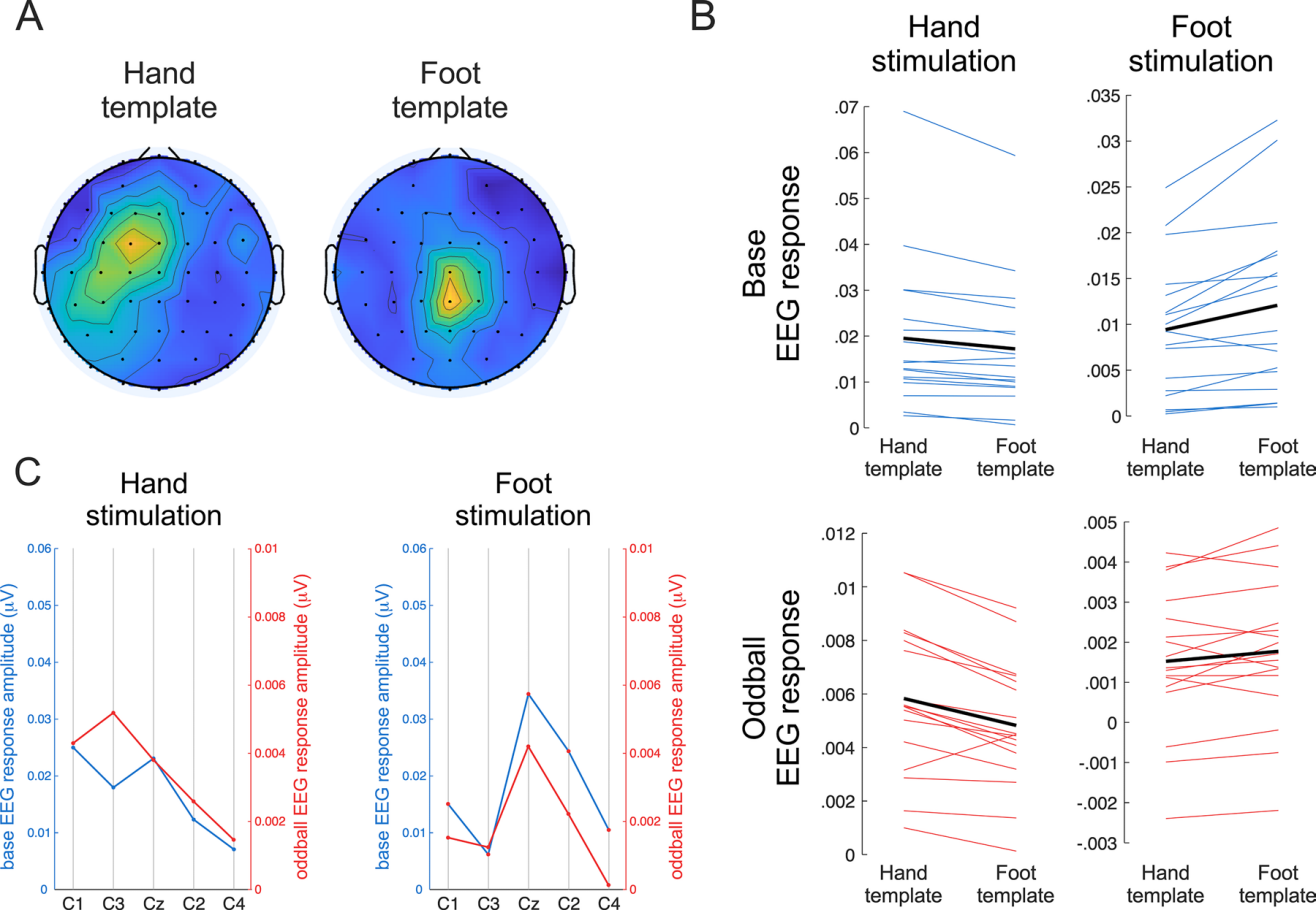
Topographic test for somatotopy in the spectrotemporal contrast condition. (A) Weighted channel templates for hand and foot stimulation, generated using the base response to the frequency/intensity contrast. (B) The left and right graphs show the responses to hand and foot stimulation, while the upper and lower graphs show the responses to the base and oddball EEG responses. Each graph compares the amplitude values weighted using the hand versus foot template. The thin blue and red lines display the data for each participant, and the thick black line shows the average across participants. Note that both for the base responses and the oddball responses, amplitude values following stimulation of the hand were greater when weighted with the hand template than when weighted with the foot template, whereas amplitude values following stimulation of the foot were greater when weighted with the foot template than when weighted with the hand template. (C) Visual comparison of base and oddball EEG responses to hand and foot stimulation at central electrodes C1, C3, Cz, C2, and C4. Individual data points represent the grand average of the activity averaged across the base and oddball harmonics pre-selected in the main topographic test for somatotopy.

The repeated-measures ANOVA showed a significant interaction between stimulation site and channel template for the oddball EEG response to the spectrotemporal contrasts (F = 23.442, p < 0.001, η^2^= 0.019). Post hoc t-tests (one-sided) showed that the oddball response to stimulation of the hand was greater when weighted with the hand template than when weighted with the foot template (p < 0.001, Cohen’s d = 1.098), and that the oddball response to stimulation of the foot was greater when weighted with the foot template than when weighted with the hand template (p = 0.022, Cohen’s d = 0.532) (Fig. 5).

Visual inspection of activity at a transversal subset of central electrodes (C1, C3, Cz, C2, and C4) confirmed that the topographical pattern of hand and foot base and oddball responses to the spectrotemporal sequences were maximal over contralateral central electrodes for hand stimulation (C1, C3), and maximal over the midline (Cz) for foot stimulation (Fig. 5C).

### 3.3 Behavioral results

Data from one subject are missing from the behavioral analysis due to issues with saving of behavioral responses. On average, participants reported perceiving the contrast within the sequence on 69.9% (SD: 28.0%) and 69.1% (SD: 28.9%) of the frequency/intensity contrast condition trials, for hand and foot stimulation, respectively. For the spectrotemporal contrast condition, participants reported perceiving the contrasts on 34.0% (SD: 24.7%) and 41.4% (SD: 28.1%) for hand and foot stimulation, respectively. A repeated measures ANOVA found no main effect of sequence on contrast detection within the spectrotemporal sequences for hand (F = 0.789, p = 0.598) nor for foot (F = 0.829, p = 0.566) stimulation.

**Fig. 6.**
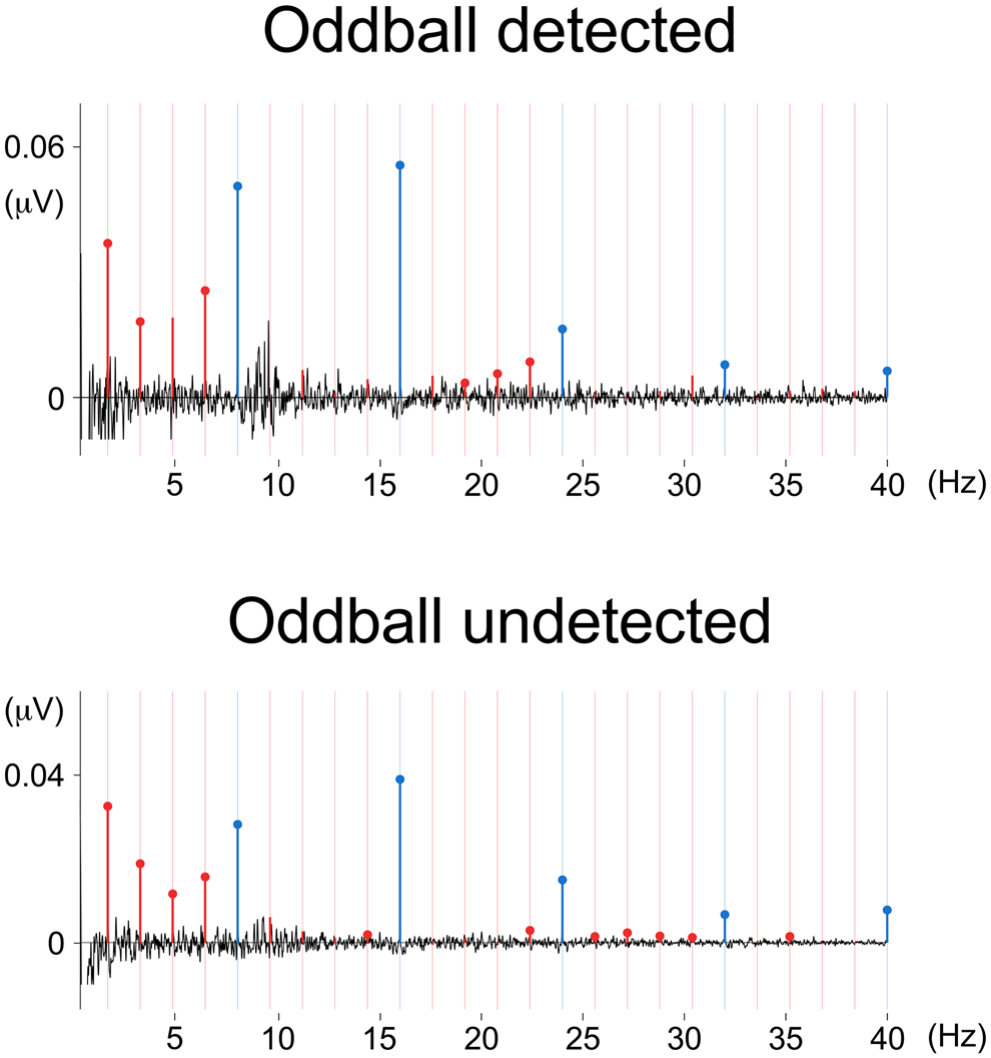
Baseline-subtracted EEG frequency spectra for detected and non-detected spectrotemporal contrast sequences, averaged across participants and EEG channels. Harmonics are color coded, with base responses depicted in blue, and oddball responses depicted in red. Filled dots depict harmonics with amplitude values significantly higher than zero (t-test, right-tailed). Vertical bars represent expected base and oddball frequencies.

To further assess whether explicit contrast perception in the spectrotemporal condition was related to the magnitude of the observed EEG responses, the pre-processed EEG trials of each participant following hand stimulation were categorized based on whether the participants had detected the contrast (two categories: detected and non-detected) and analyzed separately as described in Sections 2.6.3 and 2.6.4. For both detected and non-detected trials, we observed a significant base response (detected: mean 0.027 µV; SD = 0.0153; p < 0.001; non-detected: mean = 0.019 µV; SD = 0.013; p < 0.001) and, most importantly, a significant oddball response (detected: mean = 0.007 µV; SD = 0.008; p = 0.0036; non-detected: mean = 0.005 µV; SD = 0.003; p < 0.001) (Fig. 6). A paired-sample t-test revealed no significant differences in the magnitude of the oddball responses collected in detected versus non-detected trials (p = 0.506; N = 13 participants with both detected and non-detected trials), indicating that presence of an oddball response to the spectrotemporal contrast did not require conscious perception of the contrast.

## 4 Discussion

With this experiment, we aimed to characterize the cortical responses to fine-grained changes in the spectral pattern of vibrotactile textures (two different random noise vibrations matched in terms of average intensity, average spectral content and duration), and test the involvement of S1 by comparing these responses following stimulation of the hand and foot.

The spectrotemporal contrasts induced significant base responses (8 Hz and harmonics) and oddball responses (1.6 Hz and harmonics). When stimulating the right index finger, both responses were localized over contralateral parietal and frontal regions, compatible with the hand representation in S1. In contrast, when stimulating the foot sole, both responses were localized over central midline electrodes, consistent with the foot representation in S1 (Heid et al., 2020;Langdon et al., 2011;Moungou et al., 2016;Snyder, 1992;Tobimatsu et al., 1999).

To further investigate the cortical origin of spectrotemporal contrast responses elicited by stimulation of the hand and foot, we implemented a simple approach to assess how closely their topographies matched—across all channels—the topographical distribution of the base response to hand and foot stimulation using the frequency–intensity contrast, used as a functional localizer of activity originating from the hand and foot representations within S1, respectively. The analysis showed that the topography of the oddball response to spectrotemporal contrasts delivered onto the hand matched more closely the S1 hand representation as compared with the S1 foot representation, while the opposite was observed when stimuli were delivered onto the foot. Taken together, and without excluding a contribution of cortical areas outside S1, these results indicate an involvement of S1 in the processing of fine-grained contrasts in the pattern of complex vibrations such as those elicited during the tactile exploration of textures. Interestingly, despite their topography being highly similar, the base responses to the frequency/intensity sequences were overall lower than the base responses to the spectrotemporal sequences. While we did not expect such differences in the magnitude of activation, it is possible that these results are linked to the frequencies used for the standard and oddball stimuli in the frequency/intensity contrast condition. Indeed, seminal work has shown that the detection threshold of vibrotactile stimuli across different frequencies follows a U-shaped curve, being lowest at around 200 Hz (Mountcastle et al., 1972), corresponding to the frequency of the oddball stimulus used in the current experiment. Thus, it is possible that a stronger response to the oddball stimulus within the frequency/intensity contrast condition led to a weakening of the response to the base stimuli. Furthermore, in the frequency/intensity contrast condition, the response at the fundamental frequency of 8 Hz was not significant. This is not necessarily surprising, as the sinusoidal periodic stimulation is not expected to elicit an EEG response that is sinusoidal, due to the non-linearity of the system. Instead, the response is expected to project onto a number of harmonics of the periodic repetition rate when analyzed in the frequency domain, with a distribution of amplitudes over these harmonics depending on the shape of the periodic response (Chemin et al., 2018;Lenc et al., 2024). Furthermore, in our experiment, the sharp ramps at the onset and at the offset of each tactile event likely generated strong onset and offset responses within rapidly adapting mechanoreceptors, which could partly explain the larger peak observed at 16 versus 8 Hz.

Interestingly, the somatotopical scalp topography of the oddball response to spectrotemporal contrasts was present across all harmonic frequencies, indicating a contribution of S1 to both the slower and the faster EEG signals triggered by the oddball stimuli.

The standard and oddball stimuli constituting the spectrotemporal sequences were matched in terms of their average intensity and spectral content. However, they varied in terms of both their envelope and their spectral time course. A supplementary analysis was thus conducted to evaluate whether the observed oddball responses to the spectrotemporal sequences could have been driven solely by differences in envelope time course (Rossion et al., 2015). This analysis revealed that one of the spectrotemporal sequences exhibited a much stronger envelope dissimilarity than the other sequences, and that the oddball EEG response to that specific sequence was greater than the EEG responses to the other sequences, suggesting a contribution of envelope dissimilarity to the elicited EEG response. However, the analysis also showed that the oddball EEG response was present in each of the seven other spectrotemporal sequences, and that there was no significant relationship between envelope dissimilarity and the oddball EEG response across these seven sequences, indicating that the EEG response to the contrast was not solely driven by envelope dissimilarity. Such*a posteriori*exploration of the respective contributions of envelope versus spectrotemporal contrast across a limited number of sequences should be interpreted cautiously. Future studies are thus necessary to further establish the specific features contributing to the spectrotemporal oddball response. One approach could be to employ sequences where standard stimuli would be a random sequence of different natural textures differing greatly in terms of their low-level properties such as intensity and frequency content, while the oddball stimulus would correspond to the same texture or texture category repeated throughout the sequence—possibly also using scrambled versions of the stimuli (seeDe Keyser et al., 2018).

The magnitude of oddball responses to the spectrotemporal sequences when participants explicitly perceived the contrasts was not significantly different from the magnitude of oddball responses to the spectrotemporal sequences when the contrast was not perceived, suggesting that emergence of an oddball EEG response to the spectrotemporal contrasts did not require conscious perception of that contrast. Confirming that periodic oddball responses to fine-grained vibrational contrasts can occur in the absence of any explicit contrast perception will require further psychophysical studies. Indeed, in this experiment, participants were simply asked to report, at the end of each trial, whether they had perceived a contrast within the sequence, and no further controls such as sequences without oddball stimuli were implemented.

In conclusion, we show that minute and often unperceived variations in the spectrotemporal content and envelope of complex vibration patterns similar to those generated during the tactile exploration of textures generate a differential response within the contralateral S1. This finding indicates that fine-grained tactile texture discrimination involves or modulates S1 (or upstream subcortical structures relaying somatosensory input to S1). Most interestingly, distinguishing the oddball stimuli from the standard stimuli within the spectrotemporal sequences required to build a representation of the temporal pattern of vibrotactile stimuli (i.e., frequency content over time or intensity over time), indicating that S1 may have the ability to build such complex representations. Thus, similarly to the mechanisms underlying change-specific ERPs such as the P300 (Polich, 2007) or the mismatch negativity (Kekoni et al., 1997;Näätänen & Escera, 2000), over repeated presentation of the standard stimulus, a mental representation of the vibrotactile texture may be formed and maintained at the level of S1. In turn, presentation of the oddball vibrotactile texture, whose spectrotemporal features differ from that of the standard stimulus and, hence, the formed mental representation, would elicit a differential S1 response.

By demonstrating that the frequency-tagging approach, in combination with a periodic oddball paradigm, can be used to capture cortical responses to fine-grained changes in the spectrotemporal content of complex vibrotactile stimuli, our results open new avenues to study how the somatosensory system processes natural textures during dynamic touch.

## Supplementary Material

Supplementary Material

## Data Availability

The Matlab code for EEG and behavioral data analysis, stimuli design, and experimental procedure is available athttps://github.com/Espositogiulia/Vibrotactile-texture-experiment. The pre-processed EEG spectra, the behavioral data, and the Hilbert-Transformed A and B white noise stimuli necessary to perform the statistical analyses are available upon request athttps://doi.org/10.5281/zenodo.13842326.
