# Supplementary material for: Processing the fine-grained features of tactile textures involves the primary somatosensory cortex
Source: Imaging Neurosci (Camb). 2024 Oct 28;2:imag-2-00341. doi: 10.1162/imag_a_00341 (PMC12290531; doi:10.1162/imag_a_00341)
Supplement: Supplementary Material [file imag_a_00341-supp.pdf]

Supplementary Materials

Contribution of envelope dissimilarity to the spectrotemporal contrast EEG responses

First, we quantified the amplitude of the base and oddball EEG responses as described above (averaging across the same harmonics that were selected for the main analysis, and across channels using the weights from the S1 functional localizer constructed in the main analysis), but separately for each of the eight spectrotemporal sequences. We then carried out a linear regression analysis between the magnitude of the EEG responses to each spectrotemporal contrast and the index of envelope dissimilarity between the A and B stimuli, separately for each stimulation site. We hypothesized that if differences in envelope directly drove the oddball response, greater oddball responses would be observed for sequences exhibiting a stronger envelope dissimilarity.

An important feature of the stimulation envelope may be its attack segment. Therefore, the same analysis of envelope dissimilarity was performed considering only the first 50 ms of the A and B envelopes, to assess the potential contribution of envelope dissimilarity at the onsets of the A and B stimuli.

Analysis of the EEG responses to each individual spectrotemporal contrast revealed significant base and oddball responses for all eight sequences and for both stimulation sites (Supplementary Fig. 1).

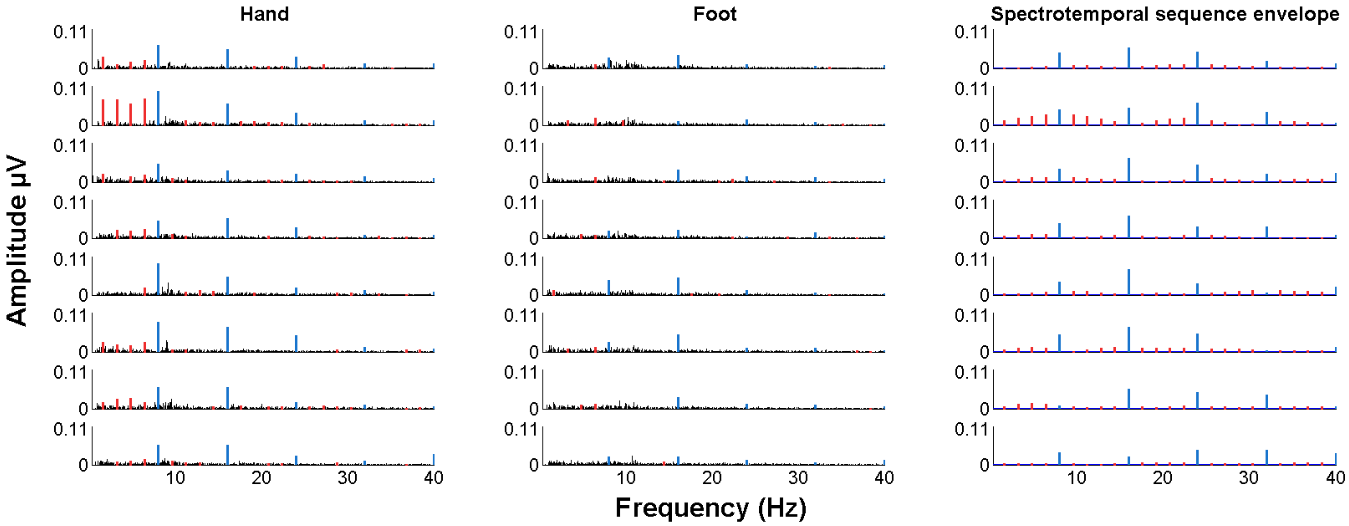

Supplementary Figure 1: Baseline-subtracted EEG frequency spectra of the responses to each spectrotemporal contrast sequence (1 to 8, top to bottom) for hand stimulation (left) and foot stimulation (middle). Harmonics with amplitude significantly higher than zero (t-test, right-tailed) are color-coded, with base responses depicted in blue, and oddball responses depicted in red. The right panel shows baseline-subtracted frequency spectra of the individual AAAAB sequence envelopes.

As shown in Supplementary Fig. 2, the envelopes of the A and B stimuli of the spectrotemporal sequence #2 exhibited a strong envelope dissimilarity as compared to the other sequences. When including this outlier sequence, the linear regression analysis showed a significant correlation between envelope dissimilarity and magnitude of the oddball EEG response for hand stimulation (R^2= 0.360, p= 0.007), and for foot stimulation (R^2= 0.049, p= 0.01). In contrast, no significant correlation was found when the spectrotemporal sequence #2 was excluded from the linear regression analysis (hand stimulation: R^2= 0.010, p= 0.271; foot stimulation: R^2= 0.003, p= 0.578).

Similarly, a significant correlation was found between envelope dissimilarity at the onset of the A and B stimuli and magnitude of the oddball EEG responses for hand stimulation (R^2= 0.182; $p<0.0001$), but not for foot stimulation (R^2= 0.012; p= 0.200). When excluding

spectrotemporal sequence #2, no significant correlation was found for either stimulation sites (hand: R^2= 0.0002; p= 0.872; foot: R^2= 0.007; p= 0.372).

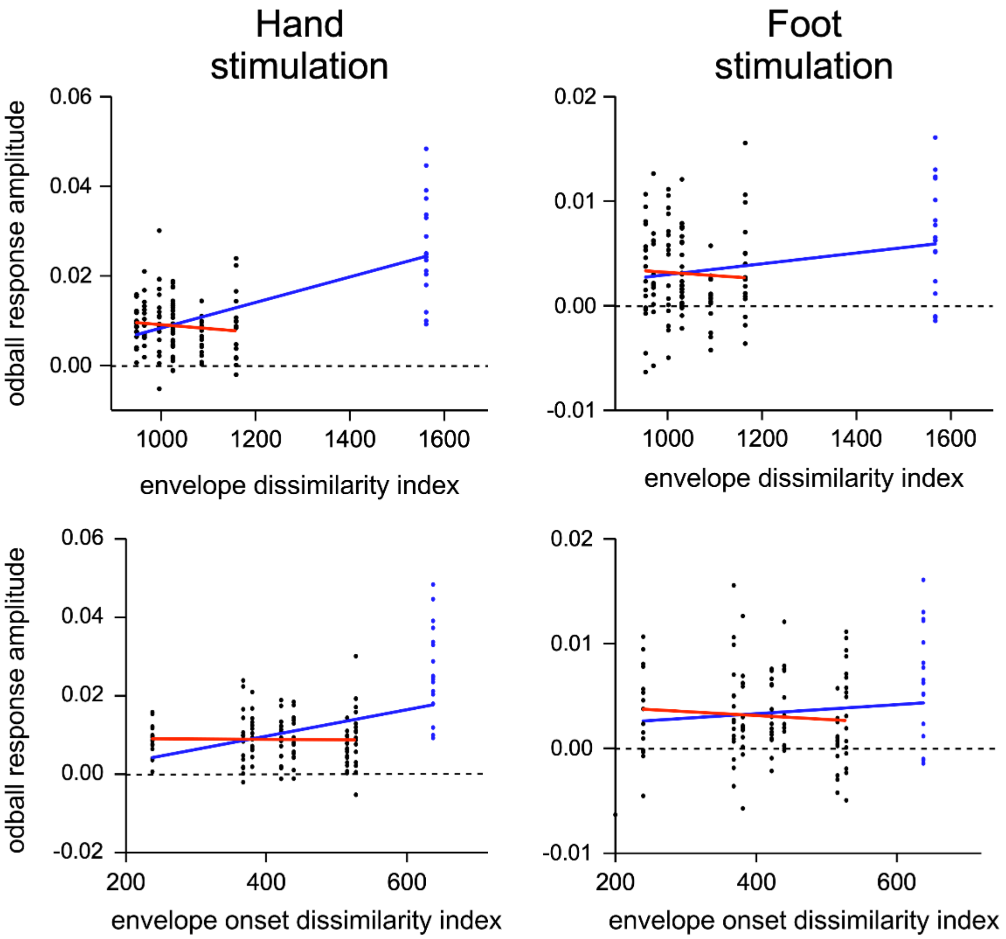

Supplementary Figure 2: Relationship between magnitude of the spectrotemporal oddball EEG responses and sequence envelope dissimilarity (top) and sequence envelope onset dissimilarity (bottom), following stimulation of the hand (left) and the foot (right). Individual dots represent single subject baseline-subtracted amplitudes, averaged across significant oddball harmonics (excluding corresponding base harmonics) and across channels, weighted according to their corresponding template. Linear regression analyses were performed including (red lines) or excluding (blue lines) spectrotemporal sequence #2 (blue dots).
